# Translation initiation factor eIF3a regulates glucose metabolism and cell proliferation *via* promoting small GTPase Rheb synthesis and AMPK activation

**DOI:** 10.1016/j.jbc.2022.102044

**Published:** 2022-05-18

**Authors:** Shijie Ma, Zizheng Dong, Yanfei Huang, Jing-Yuan Liu, Jian-Ting Zhang

**Affiliations:** 1 Department of Cell and Cancer Biology, University of Toledo College of Medicine and Life Sciences, Toledo, OH, USA; 2 Department of Medicine, University of Toledo College of Medicine and Life Sciences, Toledo, OH, USA

**Keywords:** eIF3a, protein synthesis, Rheb, AMPK, glucose metabolism, cell proliferation, ACC1, acetyl-CoA carboxylase 1, AMPK, AMP-activated protein kinase, CaMKK2, Ca^2+^/calmodulin-dependent protein kinase kinase 2, cDNA, complementary DNA, eIF3a, eukaryotic translation initiation factor 3 subunit A, FBS, fetal bovine serum, LKB1, liver kinase B1, MO25α, mouse protein 25 alpha, 2-NBDG, 2-deoxy-2-[(7-nitro-2,1,3-benzoxadiazol-4-yl) amino]-d-glucose, NP-40, Nonidet P-40, NSCLC, non–small cell lung cancer, PD, pull-down, STRAD, STE20-related kinase adaptor protein, TAK1, transforming growth factor-β–activating kinase 1

## Abstract

Eukaryotic translation initiation factor 3 subunit A (eIF3a), the largest subunit of the eIF3 complex, has been shown to be overexpressed in malignant cancer cells, potentially making it a proto-oncogene. eIF3a overexpression can drive cancer cell proliferation but contributes to better prognosis. While its contribution to prognosis was previously shown to be due to its function in suppressing synthesis of DNA damage repair proteins, it remains unclear how eIF3a regulates cancer cell proliferation. In this study, we show using genetic approaches that eIF3a controls cell proliferation by regulating glucose metabolism *via* the phosphorylation and activation of AMP-activated protein kinase alpha (AMPKα) at Thr^172^ in its kinase activation loop. We demonstrate that eIF3a regulates AMPK activation mainly by controlling synthesis of the small GTPase Rheb, largely independent of the well-known AMPK upstream liver kinase B1 and Ca^2+^/calmodulin-dependent protein kinase kinase 2, and also independent of mammalian target of rapamycin signaling and glucose levels. Our findings suggest that glucose metabolism in and proliferation of cancer cells may be translationally regulated *via* a novel eIF3a–Rheb–AMPK signaling axis.

Regulation of mRNA translation (or protein synthesis) plays an important role in controlling gene expression, and its dysregulation associates with health disorders such as cancer (1, 2), and it mainly takes place in the initiation step involving many eukaryotic translation initiation factor (eIF) complexes (3). The largest and most complicated of such complexes is eIF3, consisting of 13 subunits known as eIF3a to eIF3m (4). eIF3a, a 170-kDa protein containing three putative domains including proteasome-COP9-initiating factor 3, spectrin, and the C-terminal 10 amino acid repeat domain (5), has been shown to overexpress in many types of cancers (6, 7, 8). eIF3a has also been thought to have a noncanonical function outside the eIF3 complex to regulate translation of a subset of mRNAs by suppressing the translation of some mRNAs, whereas activating the others (9, 10). It has also been shown to associate with cancer prognosis and patient response to chemotherapy (11, 12, 13) and to regulate cancer cell proliferation (14, 15, 16, 17). Although the eIF3a function in cancer prognosis has been shown to attribute to its function in suppressing synthesis of DNA damage repair proteins in cellular response to DNA-damaging drugs and radiation (11, 18, 19), it remains to be determined how eIF3a controls cancer cell proliferation.

Glucose metabolism is primarily glycolytic even in the presence of abundant oxygen in cancer cells. Aerobic glycolysis (the Warburg effect) has been shown to confer bioenergetic advantages to proliferating cells by generating metabolic intermediates from glucose (20). The AMP-activated protein kinase (AMPK), a key player in the metabolic system, promotes glucose uptake and fast conversion of glucose to lactate (21). It is a highly conserved serine–threonine kinase complex that forms heterotrimers composed of a catalytic (α) subunit and two regulatory (β and γ) subunits, with each present in multiple isoforms, including α1, α2, β1, β2, γ1, γ2, and γ3 (22, 23). As a key regulator of cellular energy, AMPK plays a critical role in cancer cell growth and proliferation (24, 25, 26, 27, 28). AMPK activation promotes catabolic pathways to maintain cell growth and proliferation with rapid effects by directly phosphorylating metabolic enzymes and with long-term effects by regulating signal transduction and gene expression (22, 29, 30, 31, 32). Consistently, genetic or pharmacologic inhibition of AMPK activity reduces cell proliferation and induces apoptosis of cancer cells (23, 24, 31, 33).

AMPK senses and is activated by low energy levels because of energetically demanding processes through AMP binding on the γ subunit and absolutely requires phosphorylation of Thr^172^ in the activation loop of the α subunit (34). Increasing evidence suggest that AMPK is directly activated by at least two major upstream kinases, the liver kinase B1 (LKB1) and Ca^2+^/calmodulin-dependent protein kinase kinase 2 (CaMKK2) (35, 36, 37, 38). LKB1 activates AMPK under low energy conditions when intracellular AMP levels are elevated, whereas CAMKK2 activates AMPK in response to cellular calcium flux, regardless of cellular energy status. In addition, AMPK can be activated by transforming growth factor-β–activating kinase 1 (TAK1) (39) and the small GTPase Rheb (40). While TAK1 mediates tumor necrosis factor–related apoptosis-inducing ligand–induced activation of AMPK, independent of LKB1 and CAMKK2, Rheb activates AMPK in Tsc2-null cells in a mammalian target of rapamycin complex 1 (mTORC1)–independent manner. AMPK is also negatively regulated by multiple mechanisms including the serine/threonine protein kinase AKT (41), a stable complex formed by glycogen synthase kinase 3 through interactions with the AMPKβ subunit (42), the phosphorylation of AMPKα1 at Ser^485^, and the phosphorylation of AMPKα2 at Ser^491^ (43, 44).

Recently, we showed that eIF3a regulates Raptor phosphorylation at Ser^792^ (45), a direct substrate of AMPK (46), suggesting that eIF3a may regulate AMPK that in turn regulates glucose metabolism and cell proliferation. In this study, we tested this possibility and determined the mechanism of eIF3a action in controlling cell proliferation. We show that eIF3a upregulates AMPK activity and glucose metabolism possibly by controlling Rheb protein synthesis, which may mediate eIF3a regulation of cancer cell proliferation.

## Results

### eIF3a knockdown reduces AMPK activity

**Figure 1:**
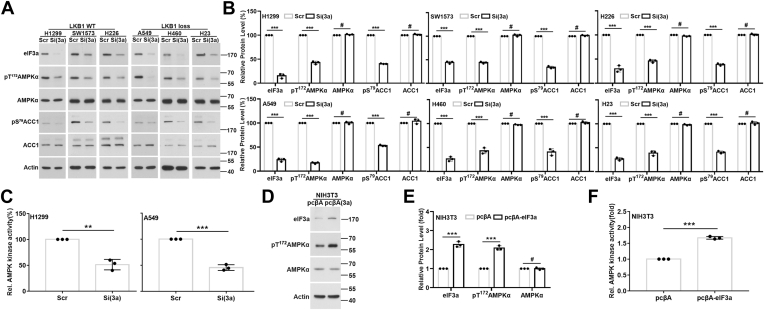
**eIF3a regulation of AMPK activity.***A* and *B*, lysates from H1299, SW1573, H226, A549, H460, and H23 cells transfected with scrambled control (Scr) or eIF3a (Si(3a)) siRNA were subjected to Western blot analyses of eIF3a, AMPKα, pT^172^AMPKα, ACC1, pS^79^ACC1, and actin loading control. *C* and *F*, lysates from H1299 and A549 cells transfected with scrambled control (Scr) or eIF3a (Si(3a)) siRNA (*C*) or from NIH3T3 cells with stable eIF3a overexpression (eIF3a) or harboring vector control (Vec) (*F*) were subjected to AMPK activity assay. *D* and *E*, lysates from NIH3T3 cells with stable eIF3a overexpression (eIF3a) or harboring Vec were subjected to Western blot analyses of eIF3a, AMPKα, pT^172^AMPKα, and actin loading control. *B* and *E*, quantifications of protein intensity in *A* and *D*, respectively. (n = 3, ∗∗*p* < 0.01, ∗∗∗*p* < 0.001). ACC1, acetyl-CoA carboxylase 1; AMPK, AMP-activated protein kinase; eIF3a, eukaryotic translation initiation factor 3 subunit A.

To test the hypothesis that eIF3a regulates AMPK activity, we took advantage of human non–small cell lung cancer (NSCLC) cell lines with (H1299, SW1573, and H226) or without (A549, H460, and H23) endogenous wildtype LKB1 and evaluated AMPK activation following eIF3a knockdown by assessing the phosphorylation of the AMPKα activation loop residue Thr^172^ and phosphorylation of its substrate acetyl-CoA carboxylase 1 (ACC1) at its Ser^79^. ACC1 is solely phosphorylated on Ser^79^ by AMPK as a direct substrate of and has been used as a marker of AMPK activation (47). As shown in Figure 1, *A* and *B*, eIF3a knockdown significantly reduced the levels of both pT^172^AMPKα and pS^79^ACC1 with little effect on the expression of total AMPKα and ACC1 in all cell lines examined regardless of their LKB1 status. We also performed an *in vitro* AMPK activity assay using lysate from H1299 and A549 cells following eIF3a knockdown. As shown in Figure 1*C*, AMPK activity was significantly reduced by eIF3a knockdown, consistent with the findings on AMPK activation and ACC1 phosphorylation analyzed using Western blot.

To ensure that the aforementioned observations were due to specific effect of eIF3a knockdown, we tested two additional siRNAs (#2 and #3) with different eIF3a-targeting sequences. As shown in Fig. S1, *A* and *B*, both siRNA#2 and #3 successfully knocked down eIF3a expression, which led to decreased level of pT^172^AMPKα and AMPK activity, similar to that by the first eIF3a siRNA.

To ensure scientific rigor and to further validate aforementioned findings, we took advantage of the stable NIH3T3 cells overexpressing eIF3a (11) and performed a reverse experiment to determine the effect of eIF3a overexpression on AMPK activity. As shown in Figure 1, *D*–*F*, the pT^172^AMPK level and AMPK activity were significantly increased in cells with eIF3a overexpression compared with the control cells harboring empty vector. Hence, eIF3a likely regulates AMPK activity regardless of the LKB1 status.

### eIF3a regulation of AMPKα1 and AMPKα2 phosphorylation and activation

**Figure 2:**
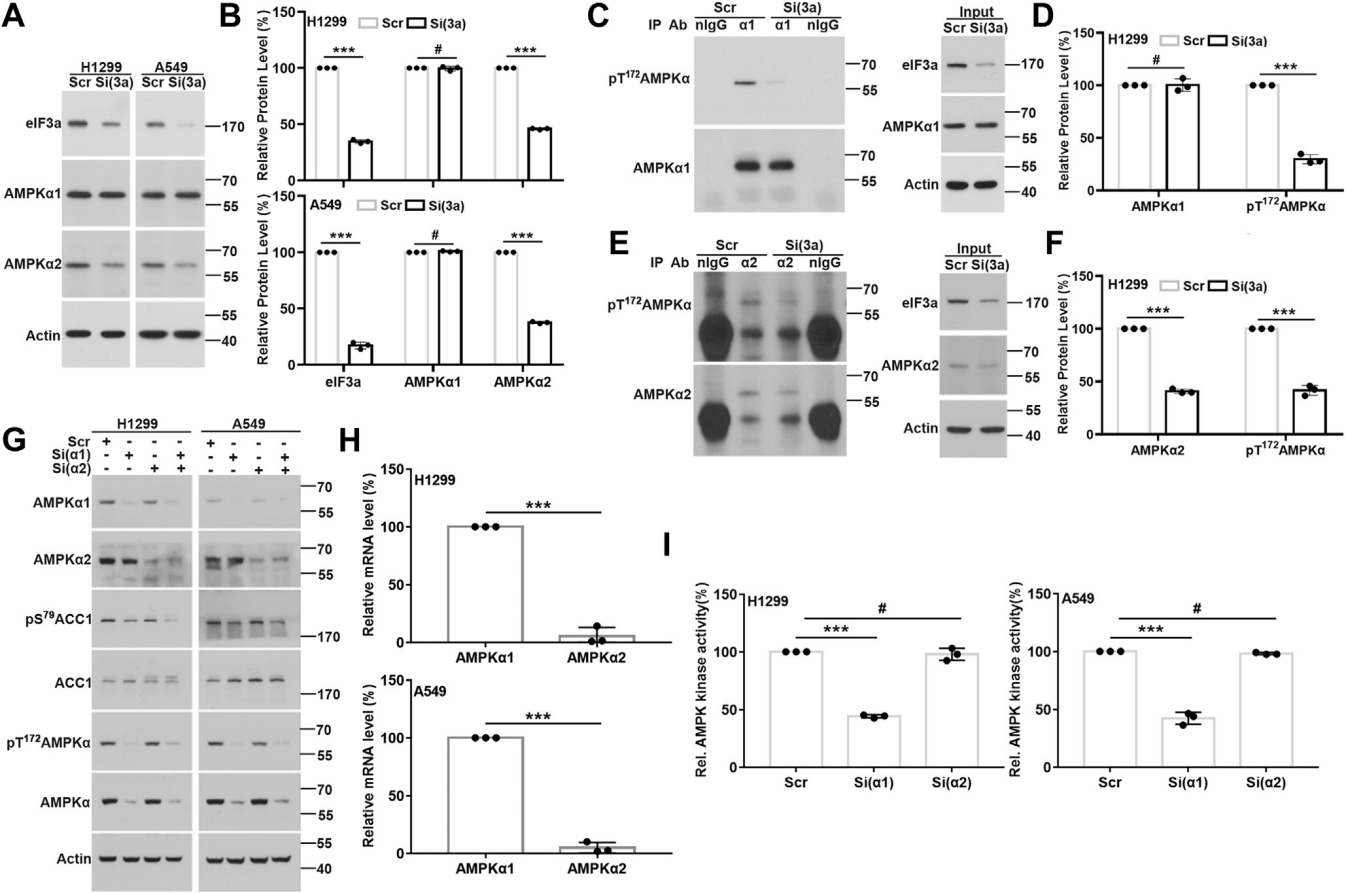
**eIF3a regulation of AMPKα1 and AMPKα2 expression.***A* and *B*, Western blot analyses of eIF3a, AMPKα1, AMPKα2, and actin loading control in H1299 and A549 cells transfected with scrambled control (Scr) or eIF3a (Si(3a)) siRNA. *C*–*F*, effect of eIF3a knockdown on AMPKα1 or AMPKα2 phosphorylation. Lysates from H1299 cells transfected with Scr or eIF3a (Si(3a)) siRNA were subjected to immunoprecipitation using AMPKα1 (*C* and *D*) or AMPKα2 (*E* and *F*) antibody or control normal IgG (nIgG) followed by Western blot analyses of pT^172^AMPKα, AMPKα1, or AMPKα2 in the precipitate and input control. *B*, *D*, and *F*, quantifications of protein intensity in *A*, *C*, and *E*, respectively. *G*, Western blot analysis of AMPKα1, AMPKα2, AMPKα, pT^172^AMPKα, ACC1, pS^79^ACC1, and actin loading control in H1299 and A549 cells transfected with Scr, AMPKα1 (Si(α1)), AMPKα2 (Si(α2)) siRNA, or both AMPKα1 and AMPKα2 siRNAs. *H*, real-time RT–PCR analyses of AMPKα1 and AMPKα2 in H1299 and A549 cells. *I*, AMPK activity in H1299 and A549 cells transfected with Scr, AMPKα1 (Si(α1)), or AMPKα2 (Si(α2)) siRNA (n = 3, ∗∗∗*p* < 0.001). ACC1, acetyl-CoA carboxylase 1; AMPK, AMP-activated protein kinase; eIF3a, eukaryotic translation initiation factor 3 subunit A.

Because there are two AMPKα isoforms (α1 and α2) and the antibody against pT^172^AMPKα does not differentiate them, we next determined which isoform is inhibited by eIF3a knockdown using Western blot analysis. As shown in Figure 2, *A* and *B*, eIF3a knockdown caused a reduction in the level of AMPKα2 protein but not that of AMPKα1 in both H1299 and A549 cells. We also determined mRNA levels of AMPKα1 and AMPKα2 using real-time RT–PCR following eIF3a knockdown in these cells. Consistent with the change in the protein level, the mRNA level of AMPKα2 but not AMPKα1 was reduced by eIF3a knockdown (Fig. S2).

Finally, we determined the relative contribution of AMPKα1 and AMPKα2 to the total AMPKα activity that are inhibited by eIF3a knockdown. For this purpose, AMPKα1 and AMPKα2 were immunoprecipitated using their respective specific antibodies from H1299 cells with eIF3a knockdown and analyzed for the phosphorylation status of Thr^172^ using Western blot. As shown in Figure 2, *C*–*F*, eIF3a knockdown reduced the level of both pT^172^AMPKα1 and pT^172^AMPKα2 in the immunoprecipitate.

The aforementioned finding that total AMPKα2 but not AMPKα1 was reduced while the phosphorylation and activation of both AMPKα1 and AMPKα2 were reduced by eIF3a knockdown suggests that AMPKα2 may contribute to eIF3a regulation of AMPK activity. To test this possibility, we first determined if AMPKα2 plays a major role in total AMPKα phosphorylation and activation since AMPKα1 and AMPKα2 can phosphorylate and activate each other (43, 44). For this purpose, we determined the level of pT^172^AMPKα and pS^79^ACC1 in H1299 and A549 cells after knocking down AMPKα1, AMPKα2, or both. As expected, the AMPKα1 or AMPKα2 protein level was reduced by their respective siRNAs compared with the cells transfected with scrambled control siRNA (Fig. 2*G*). Interestingly, the levels of pT^172^AMPKα, total AMPKα, and pS^79^ACC1 were downregulated by AMPKα1 but not AMPKα2 knockdown (Fig. 2*G*). This finding is peculiar and suggests that the abundance of AMPKα1 and AMPKα2 may differ in these cells.

To determine the relative abundance of AMPKα1 and AMPKα2, we analyzed their relative mRNA levels in H1299 and A549 cells using real-time RT–PCR. As shown in Figure 2*H*, AMPKα2 mRNA represents only 5% of that of AMPKα1. Furthermore, the total AMPK activity was inhibited when AMPKα1 but not AMPKα2 was depleted (Fig. 2*I*). Thus, the abundance of AMPKα2 is likely much less than that of AMPKα1 in these cells and possibly plays a minor role in overall AMPK activity. Together with the aforementioned findings, we conclude that AMPKα2 unlikely mediates eIF3a regulation of AMPK activity because of its low abundance although eIF3a regulates its expression and that AMPKα1 may be responsible for eIF3a regulation of the overall AMPK activity.

### eIF3a does not affect AMP/ATP ratio

It has been shown that AMPK activity is regulated by AMP/ATP ratio, and the increase in the AMP/ATP ratio triggers AMPK activation and phosphorylation of its downstream targets (48). We, thus, determined the effects of eIF3a knockdown on the intracellular AMP and ATP levels in H1299 cells, which may mediate eIF3a regulation of AMPK activation. As shown in Fig. S3, eIF3a knockdown did not change the levels of intracellular AMP and ATP or the AMP/ATP ratio. Thus, AMP/ATP ratio unlikely mediates eIF3a regulation of AMPK activation.

### eIF3a regulates AMPK not *via* CAMKK2 or TAK1

**Figure 3:**
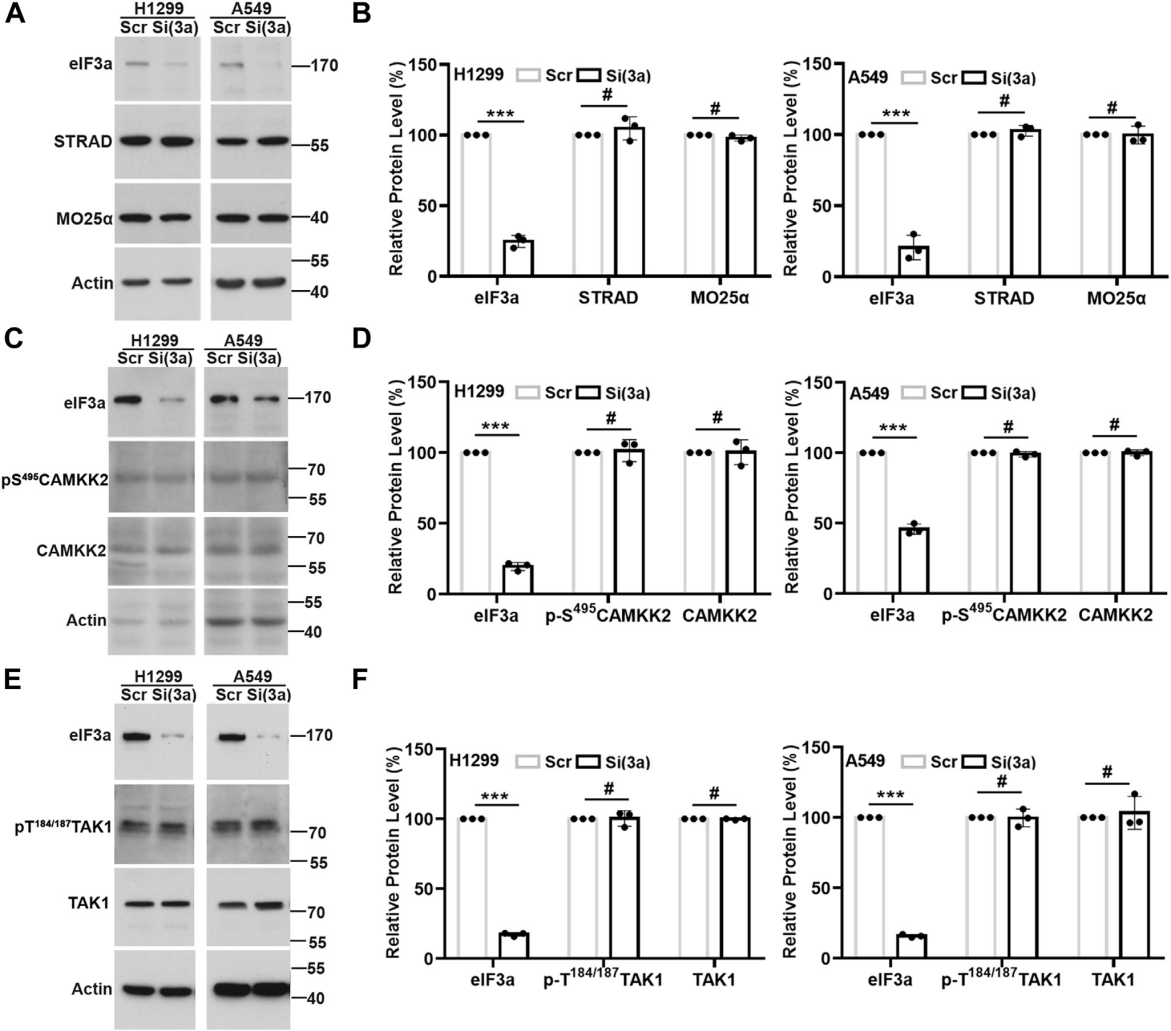
**eIF3a regulation of STRAD, MO25α, CAMKK2, and TAK1.** Western blot analyses of STRAD and MO25α (*A* and *B*), CAMKK2 and pS^495^CAMKK2 (*C* and *D*), TAK1 and pT^184/187^TAK1 (*E* and *F*) in H1299 and A549 cells transfected with scrambled control (Scr) or eIF3a (Si(3a)) siRNA. eIF3a was tested to ensure its knockdown, and actin was used as a loading control. *B*, *D*, and *F*, quantifications of protein intensity in *A*, *C*, and *E*, respectively (n = 3, ∗∗∗*p* < 0.001). CAMKK2, Ca^2+^/calmodulin-dependent protein kinase kinase 2; eIF3a, eukaryotic translation initiation factor 3 subunit A; MO25α, mouse protein 25 alpha; STRAD, STE20-related kinase adaptor protein; TAK1, transforming growth factor-β–activating kinase 1.

To understand the mechanism of eIF3a regulation of AMPK activity, we next studied other members of the LKB1 complex, the pseudokinase STRAD (STE20-related kinase adaptor protein) and the scaffolding protein MO25α (mouse protein 25 alpha), as well as CAMKK2 and TAK1, known enzymes in activating AMPK using LKB1-proficient H1299 and LKB1-deficient A549 cells. As shown in Figure 3, *A* and *B*, eIF3a knockdown did not change the level of these proteins in both H1299 and A549 cells. Because phosphorylation of the highly conserved Ser^495^ within the CaM-binding sequence impairs Ca^2+^–CaM binding and activation of CAMKK2 (49) and phosphorylation of Thr^184/187^ is an essential step for complete TAK1 kinase activation (50), we also determined if eIF3a knockdown affects the phosphorylation of these residues in CAMKK2 and TAK1. As shown in Figure 3, *C*–*F*, eIF3a knockdown did not change the level of pS^495^CAMKK2 and pT^184/187^TAK1, suggesting that CAMKK2 and TAK1 may not mediate eIF3a regulation of AMPK.

Since AMPK activation is highly dependent on glucose levels (51), we tested the possibility that CAMKK2 or TAK1 may mediate eIF3a regulation of AMPK activity under different glucose conditions. As shown in Fig. S4, increasing or decreasing glucose level did not influence the effect of eIF3a knockdown or overexpression on the level of CAMKK2, pS^495^CAMKK2, TAK1, and pT^184/187^TAK1. These findings together eliminate the possible involvement of CAMKK2 and TAK1 in eIF3a regulation of AMPK. It is noteworthy, however, that eIF3a knockdown reduced LKB1 expression and that LKB1 overexpression rescued eIF3a knockdown–induced pT^172^AMPK reduction in the LKB1-proficient H1299 cells (Fig. S5, *A*–*C*). Thus, LKB1 likely participates in mediating eIF3a regulation of AMPK activity only in LKB1-proficient cells.

### eIF3a regulates AMPK *via* Rheb

**Figure 4:**
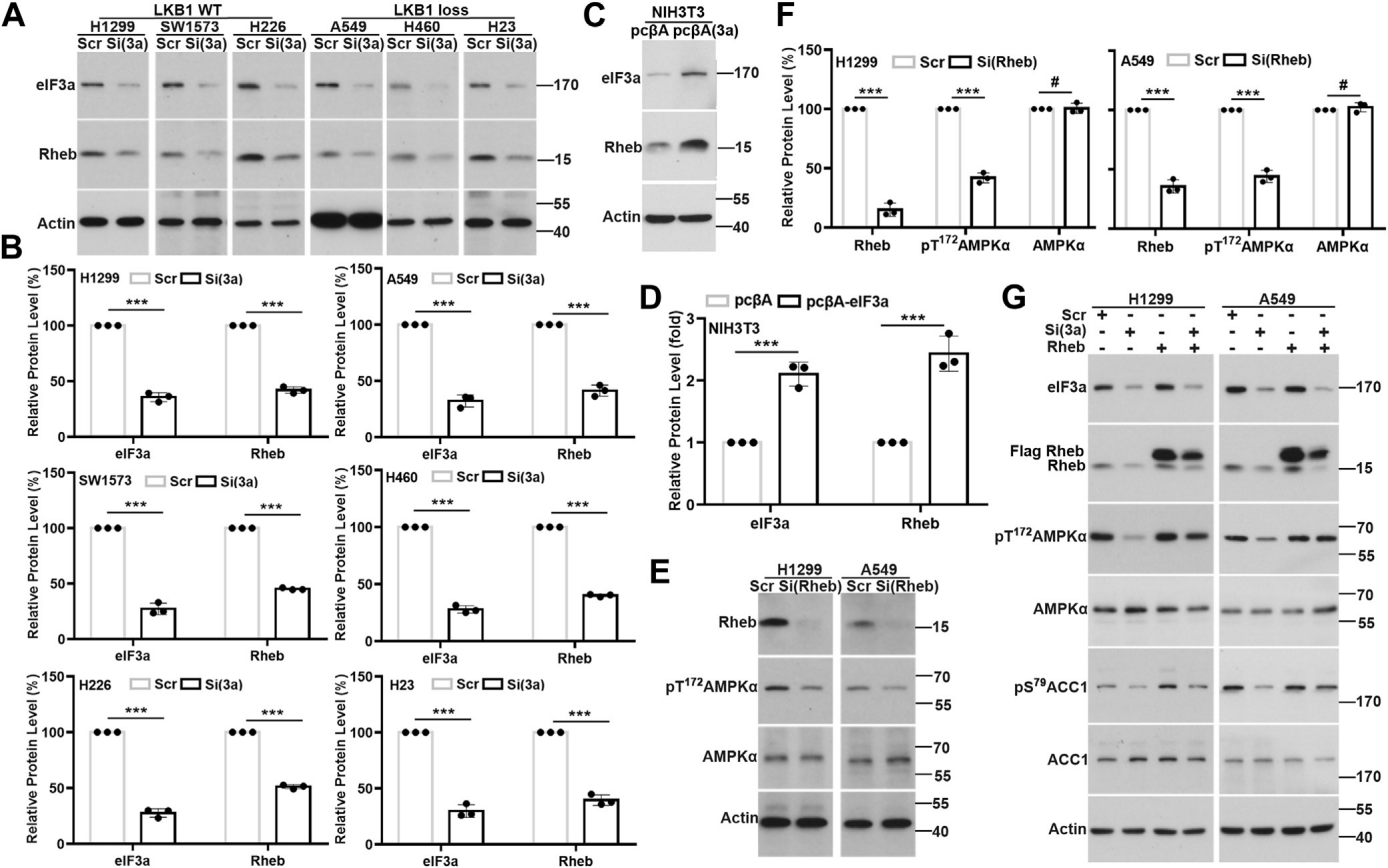
**Rheb mediates eIF3a regulation of AMPK activation.***A*–*D*, Western blot analyses of eIF3a, Rheb, and actin loading control in H1299, SW1573, H226, A549, H460, and H23 cells transfected with scrambled control (Scr) or eIF3a (Si(3a)) siRNA (*A* and *B*) and NIH3T3 cells with stable eIF3a overexpression (eIF3a) or harboring vector control (Vec) (*C* and *D*). *B* and *D*, quantification of eIF3a and Rheb from *A* and *C*, respectively (n = 3, ∗∗∗*p* < 0.001). *E* and *F*, Western blot analyses of Rheb, AMPKα, pT^172^AMPKα, and actin loading control in H1299 and A549 cells transfected with Scr or Rheb (Si(Rheb)) siRNA. *F*, quantification of eIF3a, Rheb, AMPKα, and pT^172^AMPKα from *E* (n = 3, ∗∗∗*p* < 0.001). *G*, Western blot analysis of eIF3a, Rheb, AMPKα, pT^172^AMPKα, ACC1, pS^79^ACC1, and actin loading control in H1299 and A549 cells transfected with Scr or eIF3a (Si(3a)) siRNA together with or without the plasmid overexpressing ectopic FLAG-Rheb (Rheb(OE)). ACC1, acetyl-CoA carboxylase 1; AMPK, AMP-activated protein kinase; eIF3a, eukaryotic translation initiation factor 3 subunit A.

Although the aforementioned findings suggest that LKB1 may mediate eIF3a regulation of AMPK activation in LKB1-proficient cells, there should be an alternative pathway in LKB1-deficient cells. Previously, it has been reported that Rheb controls cancer cell proliferation by regulating AMPK (40). Thus, we tested the possibility that eIF3a may work through Rheb in the LKB1-deficient cells. Interestingly, eIF3a knockdown reduced Rheb protein level in all six LKB1-proficient and LKB1-deficient cells (Fig. 4, *A* and *B*). eIF3a knockdown using two other independent siRNAs targeting eIF3a (#2 and #3) also resulted in reduction in Rheb protein level (Fig. S6). Furthermore, eIF3a overexpression in NIH3T3 cells increased the level of Rheb protein (Fig. 4, *C* and *D*). Together, these results suggest that eIF3a likely regulates Rheb expression in both LKB1-proficient and LKB1-deficient cells.

Next, we determined if Rheb possibly regulates AMPKα phosphorylation in LKB1-proficient and LKB1-deficient cells using siRNA to knock down Rheb expression and Western blot analysis of pT^172^AMPKα. As shown in Figure 4, *E* and *F*, Rheb knockdown reduced the level of pT^172^AMPKα in both LKB1-proficient H1299 and LKB1-deficient A549 cells. Consistently, overexpressing ectopic Rheb rescued eIF3a knockdown–induced reduction in pT^172^AMPKα level in these cells (Fig. 4*G*). Hence, we conclude that Rheb is likely responsible for eIF3a regulation of AMPK activity in both LKB1-deficient and LKB1-proficient cells.

It has been reported that Rheb is a key protein that relays upstream signals to regulate mTORC1 activity (52), and AMPK phosphorylates the key mTORC1 component Raptor to inhibit mTORC1 signaling (46). To examine whether mTORC1 is involved in Rheb regulation of AMPK activity, we used the mTORC1 inhibitor everolimus. As shown in Fig. S7, everolimus is effective in inhibiting phosphorylation of the mTORC1 target S6K1, whereas it was unable to inhibit Rheb-induced AMPK activation. Thus, Rheb may affect AMPK function in an mTORC1-independent manner, consistent with a previous finding that Rheb activates AMPK independent of mTORC1 (40).

### eIF3a regulates the synthesis of Rheb protein

**Figure 5:**
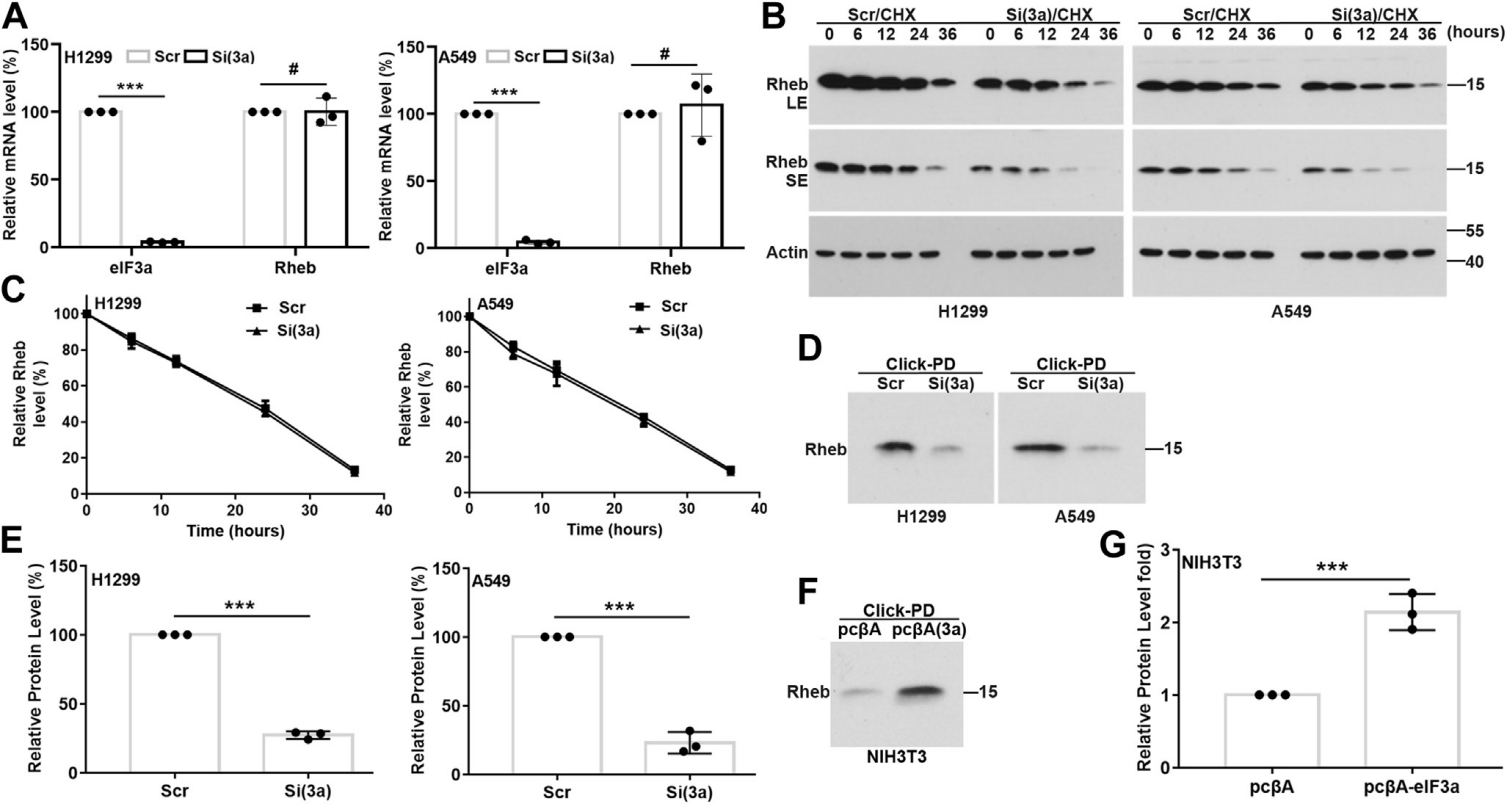
**eIF3a regulation of Rheb protein synthesis.***A*, quantitative RT–PCR analysis of Rheb mRNA levels in H1299 and A549 cells transiently transfected with eIF3a or scrambled control siRNAs (n = 3, ∗∗∗*p* < 0.001). *B* and *C*, cycloheximide (CHX)-chase analyses of Rheb protein stability in H1299 and A549 cells transfected with eIF3a or scrambled control siRNAs using Western blot. *C*, quantification of Rheb densities from *B* of three independent experiments. *D* and *E*, click-PD analysis of nascent Rheb protein in H1299 and A549 cells transiently transfected with eIF3a or scrambled control siRNAs. *E*, quantification of nascent Rheb in *D* (n = 3, ∗∗∗*p* < 0.001). *F* and *G*, NIH3T3 cells with stable eIF3a overexpression (eIF3a) or harboring vector control (Vec) were subjected to click-PD assay followed by Western blot analysis of nascent Rheb in the PD materials probed with Rheb antibody. *G*, quantification of the level of nascent Rheb protein from *F* (n = 3, ∗∗∗*p* < 0.001). eIF3a, eukaryotic translation initiation factor 3 subunit A; PD, pull down.

To determine how eIF3a regulates Rheb expression, we first determined the mRNA level of Rheb in H1299 and A549 cells following eIF3a knockdown using real-time RT–PCR. As shown in Figure 5*A*, compared with the dramatic reduction in eIF3a mRNA level, eIF3a knockdown had no effect on the mRNA level of Rheb. We next performed cycloheximide-chase and click-pull-down (PD) experiments to determine if eIF3a regulates the degradation or synthesis of Rheb protein, respectively. As shown in Figure 5, *B* and *C*, eIF3a knockdown had no effect on Rheb degradation. However, eIF3a knockdown caused drastic reduction in the level of nascent Rheb protein (Fig. 5, *D* and *E*) and, consistently, eIF3a overexpression increased the level of nascent Rheb protein (Fig. 5, *F* and *G*) as determined using click-PD. Thus, eIF3a likely regulates Rheb expression by regulating its protein synthesis.

### eIF3a regulates Rheb and AMPK independent of mTORC1 signaling and glucose level

In a previous study, we showed that eIF3a, in collaboration with HuR, regulated mTORC1 activity by controlling Raptor synthesis (45). Thus, mTOR signaling may mediate eIF3a regulation of Rheb expression although it does not mediate Rheb regulation of AMPK activation. To test this possibility, we examined if inhibiting mTORC1 using everolimus could reverse eIF3a knockdown–induced downregulation of pT^172^AMPK and Rheb expression. As shown in Fig. S8*A*, everolimus treatment significantly reduced the activation of the mTORC1 target S6K1. However, inhibiting mTORC1 failed to reverse eIF3a knockdown–induced downregulation of pT^172^AMPK and Rheb levels. Thus, mTORC1 signaling is unlikely involved in eIF3a regulation of Rheb expression and AMPK activation.

It has also been reported that AMPK activation is highly dependent on glucose levels (51). It is, thus, of interest to determine if glucose levels influence eIF3a regulation of Rheb expression and AMPK activation. For this purpose, H1299 cells with eIF3a knockdown and NIH3T3 cells with stable eIF3a overexpression along with their respective control cells were cultured in media supplemented with high or low concentration of glucose before Western blot analysis. As shown in Fig. S8, *B*–*E*, increasing or reducing glucose concentration had no effect on eIF3a regulation of Rheb expression or AMPK activation. Hence, eIF3a regulation of Rheb expression and AMPK activation is likely independent of glucose concentrations.

### eIF3 subunits and eIF3 complex in Rheb expression and AMPK activation

**Figure 6:**
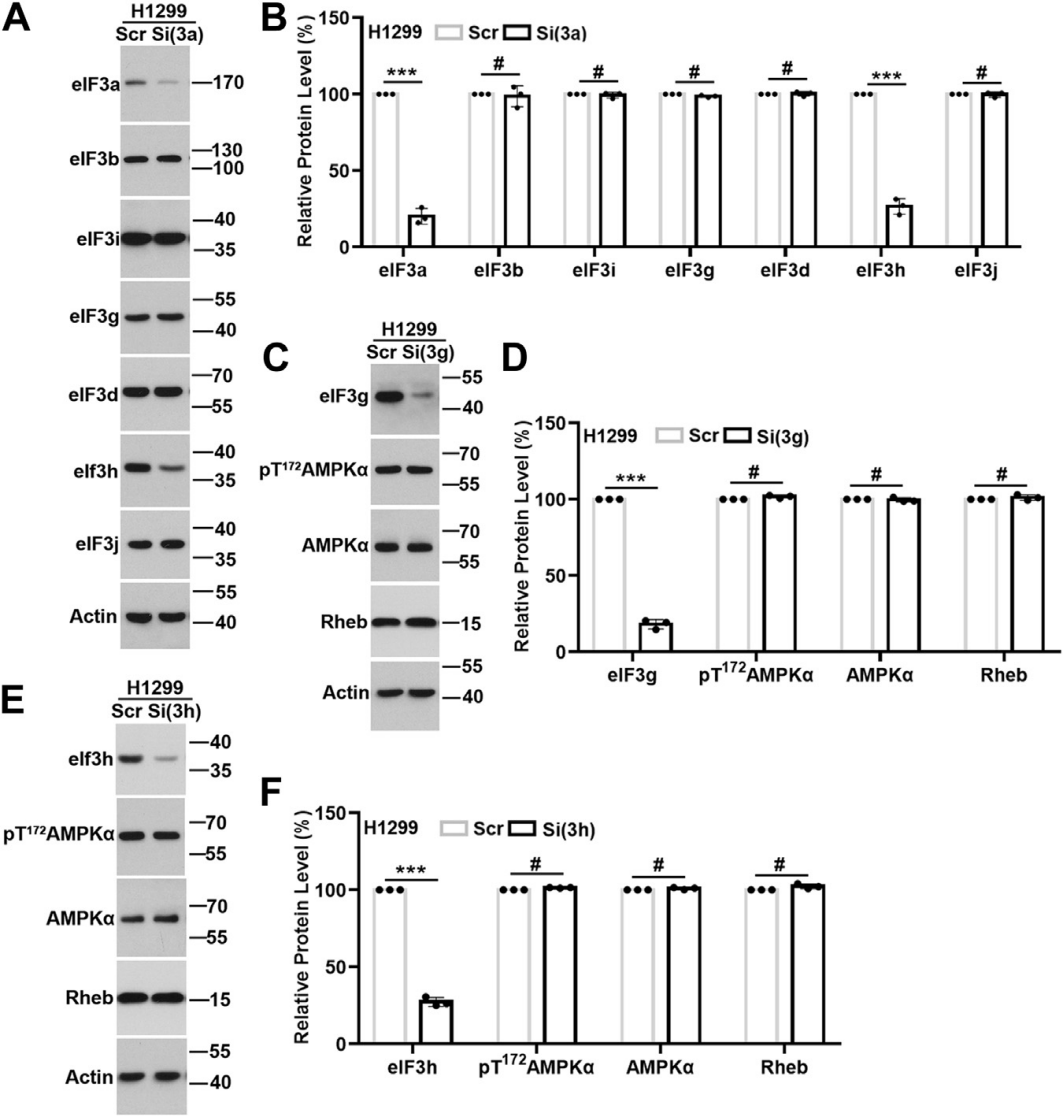
**eIF3a, other eIF3 subunits, and eIF3 complex in Rheb expression and AMPK activation.***A* and *B*, Western blot analyses of eIF3a, eIF3b, eIF3i, eIF3g, eIF3d, eIF3h, eIF3j, and actin loading control in H1299 cells transfected with scrambled control (Scr) or eIF3a siRNA (Si(3a)). *C*–*F*, Western blot analysis of eIF3g, eIF3h, AMPKα, pT^172^AMPKα, Rheb, and actin loading control in H1299 cells transfected with Scr or eIF3g siRNA (Si(3g)) (*C* and *D*) or eIF3h siRNA (Si(3h)) (*E* and *F*). *B*, *D*, and, and *F*, quantifications of protein intensity in *A*, *C*, and *E*, respectively (n = 3, ∗∗∗*p* < 0.001, #*p* > 0.05). AMPK, AMP-activated protein kinase; eIF3a, eukaryotic translation initiation factor 3 subunit A.

Because eIF3a is a subunit of eIF3(a:b:i:g) subcomplex, which, together with eIF3(c:d:e:l:k) and eIF3(f:h:m) subcomplexes, and eIF3j forms the complete eIF3 complex (53, 54), alteration of eIF3a level may disrupt this complex integrity, which in turn affect Rheb expression and AMPK activity. To eliminate this possibility, we determined the protein levels of eIF3 subunits in the eIF3(a:b:i:g) subcomplex and eIF3d and eIF3h, representative subunits in eIF3(c:d:e:l:k) and eIF3(f:h:m) subcomplexes, as well as eIF3j using immunoblot analysis following eIF3a knockdown. As shown in Figure 6, *A* and *B*, eIF3a knockdown had no effect on the level of eIF3b, i, g, d, and j. However, the level of eIF3h was significantly reduced (Fig. 6, *A* and *B*). To determine if eIF3h downregulation potentially mediates eIF3a regulation of Rheb expression and AMPK activity, we knocked down eIF3h as well as eIF3g as another representative eIF3 subunit. As shown in Figure 6, *C*–*F*, eIF3h and eIF3g knockdown in H1299 cells had no effect on the level of pT^172^AMPK, AMPK, and Rheb protein. Hence, eIF3a likely regulates Rheb expression and AMPK activity independent of other eIF3 subunits and the eIF3a complex integrity.

### eIF3a regulation of glucose metabolism and cell proliferation

**Figure 7:**
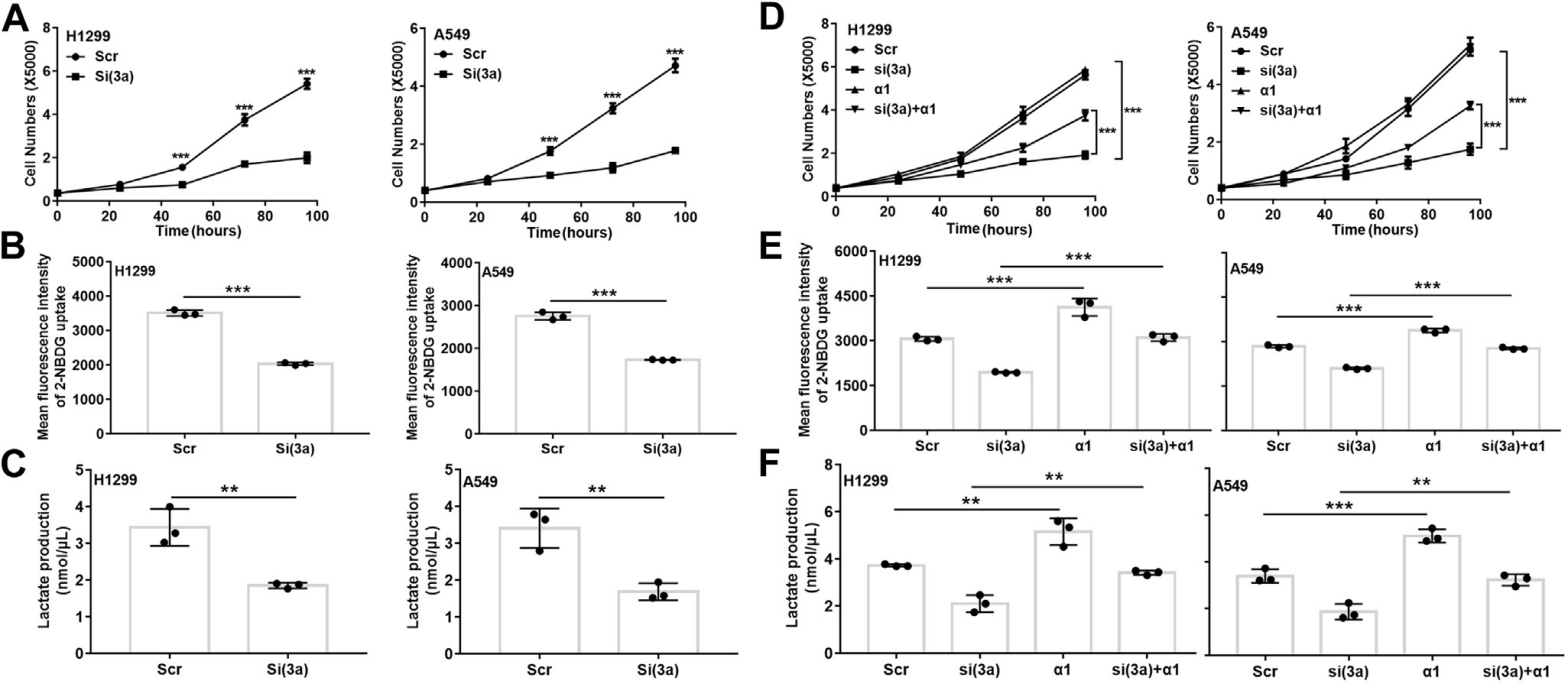
**AMPK mediates eIF3a regulation of cell proliferation and glucose metabolism.***A*, proliferation of H1299 and A549 cells transfected with scrambled control (Scr) or eIF3a (Si) siRNA. *B* and *C*, glucose uptake (*B*) and lactate production (*C*) in H1299 and A549 cells transiently transfected with eIF3a or Scr siRNAs (n = 3, ∗∗*p* < 0.01, ∗∗∗*p* < 0.001). *D*–*F*, proliferation (*D*), glucose uptake (*E*), and lactate production (*F*) of H1299 and A549 cells transiently transfected with Scr or eIF3a (Si) siRNA together with or without the plasmid overexpressing AMPKα1 (n = 3, ∗∗*p* < 0.01, ∗∗∗*p* < 0.001). AMPK, AMP-activated protein kinase; eIF3a, eukaryotic translation initiation factor 3 subunit A.

As AMPK positively regulates cell proliferation (25, 27, 31, 33), we postulated that eIF3a may regulate cell proliferation by regulating AMPK. To test this hypothesis, we first assessed the effect of eIF3a depletion on the proliferation of H1299 and A549 cells using methylene blue assay. As shown in Figure 7*A*, eIF3a knockdown significantly inhibited the proliferation of both cell lines compared with cells transfected with scrambled control siRNA, consistent with previous findings (9).

We next determined if eIF3a regulates glucose metabolism. As shown in Figure 7, *B* and *C*, eIF3a knockdown significantly decreased 2-deoxy-2-[(7-nitro-2,1,3-benzoxadiazol-4-yl) amino]-d-glucose (2-NBDG) uptake and lactate production in comparison with their respective scrambled control siRNA-transfected H1299 and A549 cells. Consistently, eIF3a overexpression increased the proliferation, 2-NBDG uptake, and lactate production in NIH3T3 cells (Fig. S9, *A*–*C*).

We finally investigated if AMPKα1 mediates eIF3a regulation of cell proliferation and glucose metabolism by performing a rescue experiment with ectopic AMPK overexpression on eIF3a knockdown in H1299 and A549 cells. As shown in Figs. 7, *D*–*F* and S9, *D*–*G*, overexpressing AMPKα1 or Rheb reversed the inhibition in cell proliferation, 2-NBDG uptake, and lactate production induced by eIF3a knockdown. Thus, eIF3a upregulation in cancer cells may turn on aerobic glucose metabolism and promote proliferation by activating the Rheb–AMPK pathway.

## Discussion

In this study, we show that eIF3a may regulate aerobic glucose metabolism and cell proliferation by regulating AMPKα1 activation *via* controlling Rheb synthesis in both LKB1-proficient and LKB1-deficient cells. However, LKB1 also contributes to eIF3a regulation of AMPKα1 in LKB1-proficient cells.

The finding that eIF3a regulates phosphorylation of AMPKα at Thr^172^ and AMPK activity is remarkable. While phosphorylation of either AMPKα1 and AMPKα2 activates AMPK and the phosphorylation of both AMPKα1 and AMPKα2 at Thr^172^ is regulated by eIF3a, AMPKα2 is much less abundant than AMPKα1 in the NSCLC cells tested in this study. Thus, AMPKα1 is likely a major contributor to AMPK activity regulated by eIF3a.

It is noteworthy that in addition to its phosphorylation, AMPKα2, not AMPKα1, was also changed at its mRNA level by eIF3a knockdown. Interestingly, knocking down AMPKα2 did not cause changes to the total AMPK activity likely because of its scarcity. However, in cells or tissues where AMPKα2 is more abundant, it could play a bigger role in eIF3a regulation of AMPK activity. To this end, it is noteworthy that AMPKα1 and AMPKα2 may have some specificity in tissue distribution, subcellular localization, and substrate selection (55, 56, 57, 58). Future studies are clearly needed to determine whether eIF3a regulation of AMPK activity is tissue or subcellular location dependent.

While both LKB1 and CaMKK2 are known major upstream activators of AMPK (37, 59), we show here that LKB1 contributes to eIF3a regulation of AMPK in only LKB1-proficient NSCLC cells, whereas CaMKK2 does not in either LKB1-proficient or LKB1-deficient cells. Although LKB1 requires the binding of MO25α and STRAD to be active and it is the LKB1–STRAD–MO25α complex that activates AMPK (60), eIF3a does not appear to regulate the expression of STRAD and MO25α. This finding is consistent with previous observations that LKB1 directly phosphorylates Thr^172^ and activates AMPKα (35, 61). In other studies, efficient activation of AMPK by LKB1 requires STRAD and MO25 subunits (60, 62). The association of LKB1 with STRADα and MO25α has also been shown to increase LKB1 kinase activity (63, 64). However, it remains unresolved how eIF3a regulates the expression of LKB1.

In addition to LKB1, we show here that Rheb is probably a major mediator in eIF3a regulation of AMPKα, especially in NSCLC cells that are LKB1 deficient. Although Rheb has been shown in a previous study to activate AMPK (40), it remains to be determined how it activates AMPK. Nevertheless, we show here that eIF3a regulates Rheb protein synthesis. It is noteworthy that other eIF3 subunits such as eIF3g and eIF3h do not regulate Rheb synthesis and AMPK activation. It is also unlikely that the eIF3a regulation of Rheb synthesis and AMPK activation is due to potential disruption of the eIF3 complex by eIF3a knockdown. Thus, eIF3a may have a noncanonical function in regulating Rheb protein synthesis and AMPK activation. Indeed, eIF3a has previously been shown to regulate translation of a subset of mRNAs possibly *via* the HuR-binding sites in their 5′-UTR or 3′-UTR (45, 65). Examination of the UTR sequence of Rheb mRNA shows that there are 4 and 36 putative HuR-binding sites in its 5′-UTR and 3′-UTR, respectively. These HuR-binding sites may contribute to eIF3a regulation of Rheb synthesis *via* HuR.

Over the past decade, a large amount of evidence has emerged in supporting the critical roles of aerobic glucose metabolism in promoting proliferation of cells in various cancer types (66, 67, 68). Furthermore, AMPK activation promotes glucose uptake by phosphorylating TBC1D1 (TBC domain family, member 1) and TXNIP (thioredoxin-interacting protein), which control the translocation and cell-surface levels of glucose transporters GLUT4 and GLUT1, respectively (69, 70). eIF3a regulation of AMPK and glucose uptake in promoting cancer cell proliferation may work through TBC1D1 and TXNIP regulation of GLUT4 and GLUT1. Although this speculation needs to be tested in future studies, our findings here provide essential evidence on translational regulation of metabolism by eIF3a in cancer cell proliferation.

## Experimental procedures

### Materials

Antibodies against AMPK, AMPKα1, AMPKα2, pT^172^AMPKα, ACC1, pS^79^ACC1, LKB1, MO25α, Rheb, eIF3h, and pS^495^CAMKK2 were obtained from Cell Signaling Technology. Antibodies against STRAD, eIF3b, eIF3i, eIF3d, eIF3j, Protein G PLUS-Agarose, and siRNAs against eIF3a, AMPKα1, AMPKα2, eIF3g, eIF3h, and Rheb were from Santa Cruz Biotechnology. CAMKK2 antibody and l-Lactate Assay Kit (catalog no.: ab65331) were from Abcam. TAK1 and pT^184/187^TAK1 antibodies were from ABclonal. Antibody against eIF3g was purchased from Invitrogen. These antibodies were validated by their respective manufacturers with information provided on their websites. The eIF3a siRNA #2 and #3 of different sequences were purchased from OriGene Technologies. The scrambled control siRNA and the Streptavidin MagneSphere Paramagnetic Particle were purchased from Applied Biosystems Ambion and Promega, respectively. The plasmid containing complementary DNAs (cDNAs) encoding human LKB1 (catalog no.: 8590), Rheb (catalog no.: 19996), and pECE-AMPKα1 (catalog no.: 69504) were from Addgene. The High-Capacity cDNA Reverse Transcription Kit, SYBR Green PCR Master Mix, 2-NBDG, and fetal bovine serum (FBS) were all from Applied Biosystems. The protease inhibitor cocktail and CycLex AMPK Kinase Assay kit (catalog no.: CY-1182) were from Roche Diagnostics and MBL International Corporation, respectively. Cell culture media were from Corning. Azidohomoalanine, biotin-PEG-4-alkyne, Tris[(1-benzyl-1,2,3-triazol-4-yl) methyl] amine, Tris(2-carboxyethyl) phosphine, CuSO_4_, and β-actin antibody were all from Sigma–Aldrich. All other chemicals were from either Fisher Scientific or Sigma–Aldrich.

### Cell lines, transient transfection, and proliferation assay

Human lung cancer cell lines H1299, SW1573, H226, A549, H460, and H23 were from American Type Culture Collection and reauthenticated using short tandem repeat on February 17, 2022. NSCLC cell lines H1299, H226, H23, and H460 were cultured in RPMI1640 containing 10% FBS. NIH3T3 cells with stable eIF3a overexpression or transfected with vector control (11) and A549 cells were cultured in Dulbecco's modified Eagle's medium containing 10% FBS. SW1573 cells were cultured in α-minimum essential medium containing 10% FBS.

For transient transfection, cells were seeded in 6-well plates and cultured for 24 h before transfection with siRNAs or plasmids using Lipofectamine RNAiMAX Regent (Invitrogen) or Lipofectamine 3000 (Invitrogen) transfection reagent according to manufacturer’s instructions. Cells were harvested for analysis 24 or 48 h after transfection.

For proliferation determination, 24 h following transfection with siRNAs or plasmids, cells were seeded in 96-well plates in triplicates followed by continuous culture for different times. Cells were then fixed with methanol and stained with methylene blue followed by determination of absorbance at 650 nm. The data were analyzed using GraphPad Prism program (GraphPad Software, Inc).

### Quantitative RT–PCR analysis

Total RNAs were purified using the PureLink RNA mini kit from Thermo Fisher Scientific according to the protocol provided by the manufacturer. First strand (cDNA) synthesis and quantitative PCR were performed using the High-Capacity cDNA Reverse Transcription Kit and SYBR Green PCR Master Mix on an Applied Biosystems 7500 PCR System. The primers used were 5′-TGATGAGGACAGAGGACCAAGAC-3’ (forward) and 5′-TCAGCATTACGCCAGGATGA-3’ (reverse) for eIF3a (65), 5′-CCTCAAGCTTTTCAGGCATC-3’ (forward), and 5′-CAAATAGCTCTCCTCCTGAGACA-3’ (reverse) for AMPKα1 (71), 5′-CAGGCCATAAAGTGGCAGTTA-3’ (forward) and 5′-AAAAATCTGTTGGAGTGCTGA-3’ (reverse) for AMPKα2 (72), 5′-GCCAATTTGTGGACTCCTACG-3’ (forward) and 5′-CCCACCATATCCAACAATTTGC-3’ (reverse) for Rheb (73), and 5′-TGGCACCCAGCACAATGAA-3’ (forward) and 5′-CTAAGTCATAGTCCGCCTAGAAGCA-3’ (reverse) for β-actin (74); all as previously described. Data were processed using the 2^−△△Ct^ formula and normalized using the internal control actin.

### Western blot analysis

Cells washed twice with ice-cold PBS were lysed in 10 mM Tris–HCl, pH 7.5, 100 mM NaCl, 1% Nonidet P-40 (NP-40), 10 mM pyrophosphate, 50 mM NaF, 2 mM EDTA, and 1 mM PMSF. After centrifugation to remove insoluble cell debris at 15,700 g for 15 min at 4 °C, the supernatant was collected for determination of protein concentrations using the Bradford reagent and separation by SDS-PAGE followed by Western blot analysis. Signals were developed using enhanced chemiluminescence, captured using X-ray film, and quantified using the ImageJ software (National Institutes of Health).

### Immunoprecipitation

For immunoprecipitation, cells were lysed as described previously, and 0.5 to 1 mg of the lysates were mixed with 20 μg of primary antibodies followed by incubation at 4 °C for 2 to 3 h with gentle agitation before mixing with 40 μl Protein G PLUS-Agarose slurry. Following incubation overnight at 4 °C with agitation, immunoprecipitates were collected by centrifugation at 400*g* for 2 min and washed six times with ice-cold lysis buffer. The precipitates were dissolved in SDS sample buffer, boiled for 5 min, and centrifuged at 400*g* for 2 min to remove insoluble materials before separation by SDS-PAGE and Western blot analysis as described peviously.

### AMPK assay

AMPK activity was measured using the CycLex AMPK Kinase Assay kit according to manufacturer’s instructions. Briefly, H1299 and A549 cells transfected with scrambled or eIF3a siRNA as described previously were lysed in 20 mM Tris–HCl, pH 7.5, 250 mM NaCl, 0.5% NP-40, 10% glycerol, 1 mM EDTA, 1 mM EGTA, 5 mM NaF, 2 mM Na_3_VO_4_, 2 mM β-glycerophosphate, 1 mM DTT, 0.2 mM PMSF, and protease inhibitor cocktail. The cell lysates were added to a plate precoated with an AMPK substrate peptide derived from mouse IRS-1 (insulin receptor substrate-1) containing Ser^789^ and incubated at 30 °C for 30 min. The phosphorylated peptides were then reacted with AS-4C4, an anti-pS^789^IRS-1 monoclonal antibody, and horseradish peroxidase–conjugated antimouse immunoglobulin in the kit. The signal was then developed using tetramethylbenzidine and detected by absorption at 450 nm.

### AMP and ATP assay

H1299 cells transfected with scrambled or eIF3a siRNA as described previously were harvested and homogenized for determination of the AMP or ATP levels with colorimetric assay using AMP and ATP assay kits from Abcam according to instructions by the manufacturer. AMP and ATP concentrations were extrapolated from standard curves and normalized to the protein content of each sample.

### Cycloheximide-chase and click-PD assays

Cycloheximide-chase assay was performed as we previously described (75). Briefly, H1299 and A549 cells transfected with scrambled or eIF3a siRNA as described previously were pretreated with 10 μM cycloheximide for various times before harvest for Western blot analysis of Rheb.

Click-PD assay was performed also as previously described (45, 65). Briefly, cells in 6-well plates were rinsed with PBS and starved in methionine-free medium for 1 h followed by culturing in methionine-free medium supplemented with azidohomoalanine for another 3 h before harvest. The cells were then lysed in 50 mM Tris–HCl, pH 7.5, 150 mM NaCl, 0.5% NP-40, 50 mM NaF, 1 mM Na_3_VO_4_, 1 mM PMSF, and 1 mM DTT followed by mixing with 0.1 mM biotin-PEG-4-alkyne, 0.04 mM Tris[(1-benzyl-1,2,3-triazol-4-yl) methyl] amine, 1 mM Tris(2-carboxyethyl) phosphine, and 1 mM CuSO_4_ with equal amount of protein. The mixtures were allowed to react for 3 h at room temperature with agitation. Nascent proteins labeled with biotin were pulled down using Streptavidin MagneSphere Paramagnetic Particles and analyzed by Western blot.

### Glucose uptake assay

Glucose uptake assay was performed using 2-NBDG as a glucose tracer as previously described (67). Briefly, 2 × 10^5^ cells per well were seeded in 6-well plates and incubated overnight before transfection with siRNAs or plasmids. Forty-eight hours after transfection, cells were deprived of glucose for 6 h and then supplemented with 50 μM 2-NBDG tracer or glucose control followed by culturing for 1 h. The cells were then harvested and washed twice with PBS before determining mean fluorescence intensity using flow cytometry.

### Lactate production assay

Lactate production was measured using the l-Lactate Assay Kit according to manufacturer’s specifications. Briefly, 2 × 10^5^ cells per well were seeded in 6-well plates overnight before transfection with siRNAs or plasmids as described previously. Cells were cultured for additional 48 h before collection of media for analyses using the kit. Each test was performed in duplicate, with output adjusted to background lactate levels in medium and normalized to total cell count.

### Statistical analysis

All statistical analyses were performed using GraphPad Prism. Each experiment was performed three times independently for statistical analyses with data presented as mean ± standard deviation. One-way ANOVA followed by Dunnett's test was used to compare more than two groups, and two-tailed Student's *t* tests were done to compare two groups. Values of *p* < 0.05 were considered statistically significant.

## Data availability

All data are contained within the article and supporting information.

## Supporting information

This article contains supporting information.

## Conflict of interest

The authors declare that they have no conflicts of interest with the contents of this article.
