# Supplementary material for: Translation initiation factor eIF3a regulates glucose metabolism and cell proliferation via promoting small GTPase Rheb synthesis and AMPK activation
Source: J Biol Chem. 2022 May 18;298(7):102044. doi: 10.1016/j.jbc.2022.102044 (PMC9207673; doi:10.1016/j.jbc.2022.102044)
Supplement: Supplemental information [file mmc1.pdf]

Supplemental Figures

Translation initiation factor eIF3a regulates glucose metabolism and cell proliferation via promoting small GTPase Rheb synthesis and AMPK activation

Shijie Ma[1], Zizheng Dong[1], Yanfei Huang[2], Jing-Yuan Liu[2], Jian-Ting Zhang[1,*]

[1] Department of Cell and Cancer Biology and [2] Department of Medicine, University of Toledo College of Medicine and Life Sciences, Toledo, Ohio

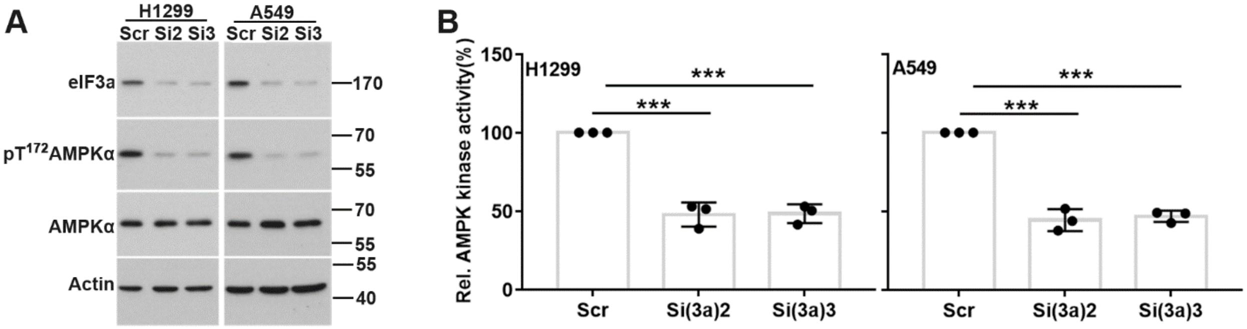

Figure S1. eIF3a regulation of AMPK activity. (A). Western blot analyses of eIF3a, AMPKα, pT^{172}AMPKα, and actin loading control in H1299 and A549 cells transfected with scrambled control (Scr) or eIF3a siRNAs (Si2 and Si3). (B). In-vitro AMPK kinase activity assay using lysates from H1299 and A549 cells transfected with scrambled control (Scr) or eIF3a siRNAs (Si2 and Si3). (n=3, ****p*< 0.001).

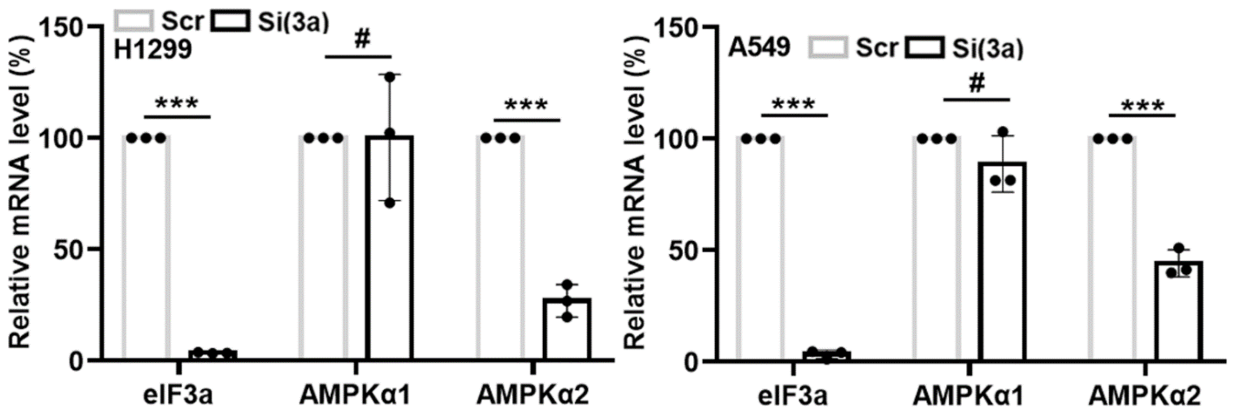

Figure S2. eIF3a regulation of AMPKα1 and AMPKα2 mRNA. Total RNAs isolated from H1299 and A549 cells transfected with scrambled control (Scr) or eIF3a (Si) siRNA were used for real-time RT-PCR analysis of eIF3a, AMPKα1 and AMPKα2. (n=3, ***p<0.001, $^{\#}p$ > 0.05).

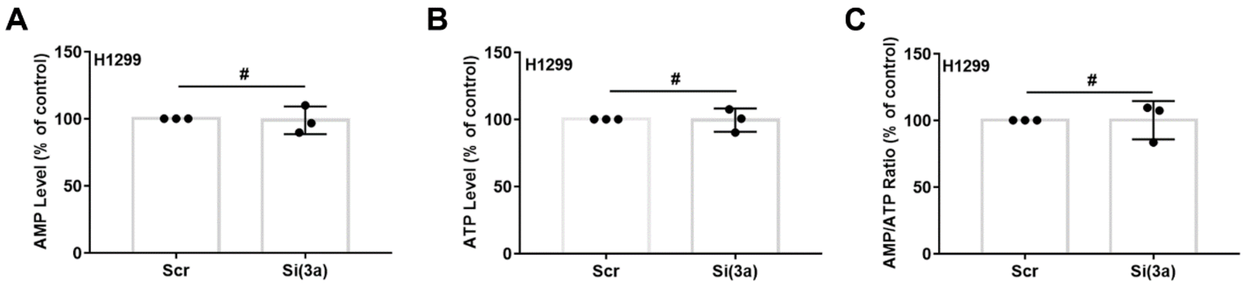

Figure S3. eIF3a regulation of AMP and ATP levels. The level of AMP (A) and ATP (B) were determined using colorimetric assay in H1299 cells following eIF3a knockdown (Si(3a)) or in control scrambled (Scr) siRNA-transfected cells. Panel C shows the AMP/ATP ratio derived from panels A and B. (n=3, $^{\#}p > 0.05$).

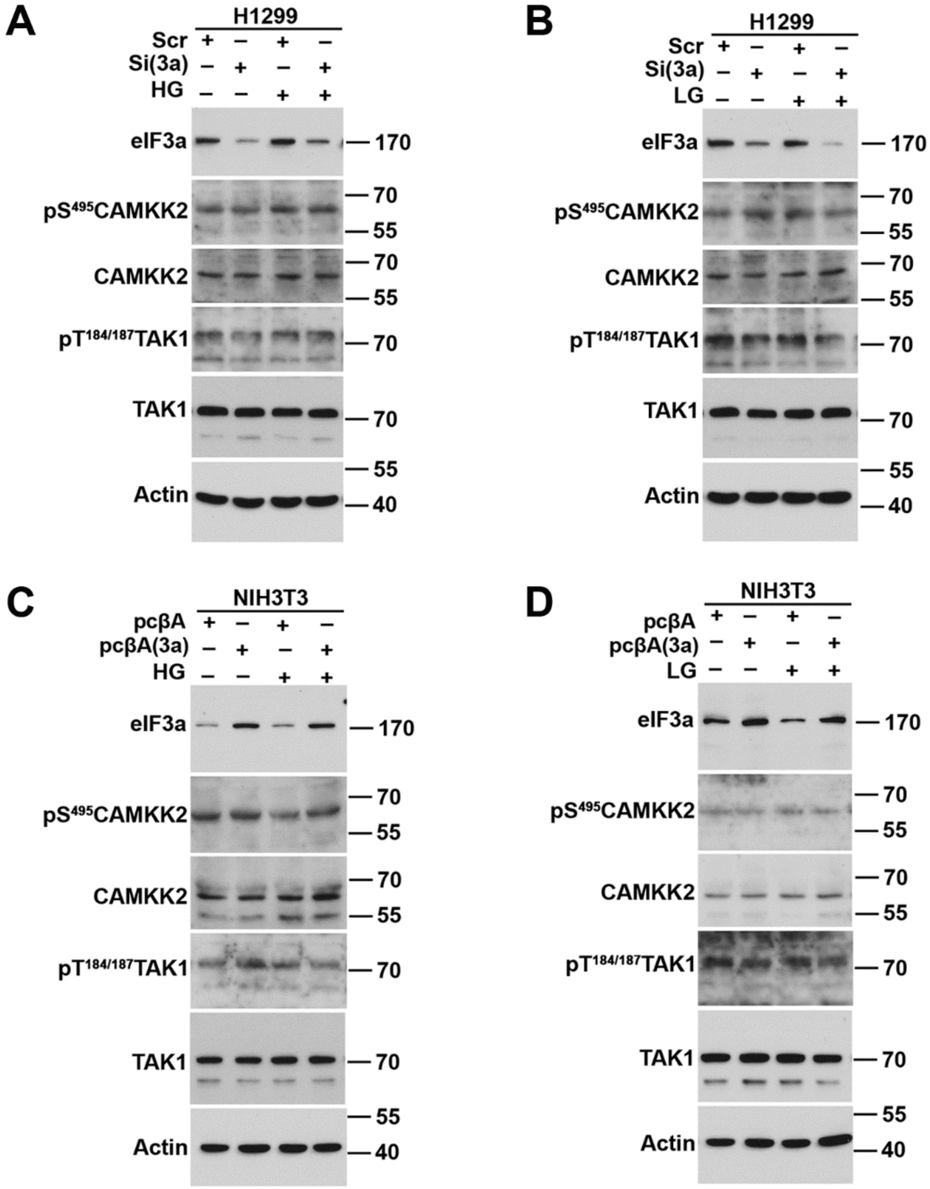

Figure S4. Role of glucose level in eIF3a regulation of CAMKK2 and TAK1. Western blot analysis of eIF3a, CAMKK2, pS^{495}CAMKK2, TAK1, pT$^{184/187}$TAK1 and actin loading control in H1299 cells (A-B) transfected with scrambled control (Scr) or eIF3a (Si(3a)) siRNA and cultured for 24 hours in the presence of 25 mM (HG) (A) or 0 mM glucose (LG) (B), or in NIH3T3 cells (C-D) with stable eIF3a overexpression (eIF3a) or harboring vector control (Vec) cultured for 24 hours in the presence of 50 mM (HG) (C) or 10 mM glucose (LG) (D).

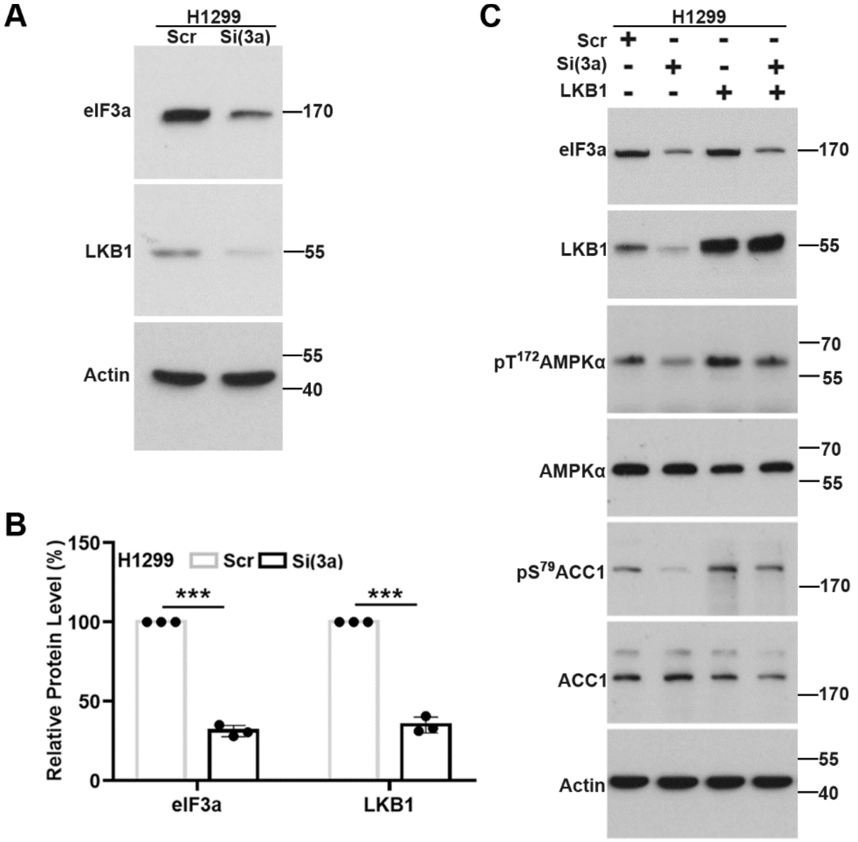

Figure S5. Role of LKB1 in eIF3a regulation of AMPK. (A-B). Western blot analysis of eIF3a, LKB1, and actin loading control in LKB1 proficient H1299 cells transfected with scrambled control (Scr) or eIF3a (Si(3a)) siRNA. Panel B shows quantifications of protein intensity in panel A (n=3, ***p<0.001). (C). Western blot analysis of eIF3a, LKB1, AMPKα, pT^{172}AMPKα, ACC1, pS79ACC1 and actin loading control in H1299 cells transfected with scrambled control siRNA (Scr), eIF3a siRNA (Si(3a)), LKB1 cDNA for overexpression (LKB1), or both eIF3a siRNA and LKB1 cDNA.

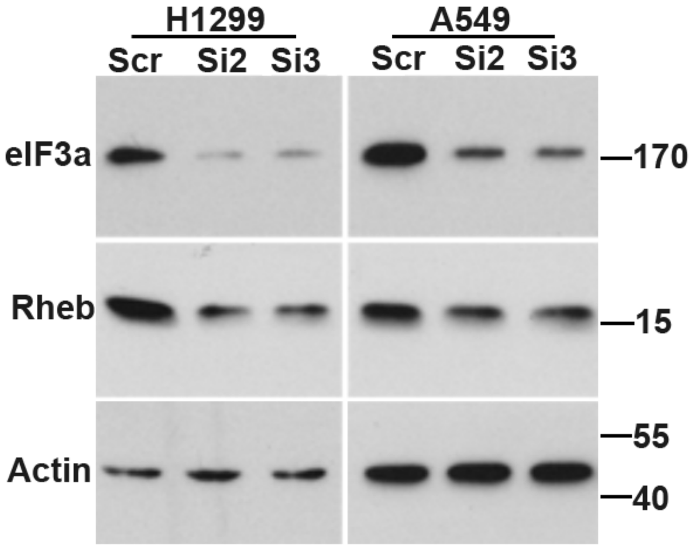

Figure S6. Effect of eIF3a knockdown on Rheb expression. Western blot analyses of eIF3a, Rheb, and actin loading control in H1299 and A549 cells transfected with scrambled control (Scr) or different eIF3a (Si2 and Si3) siRNAs.

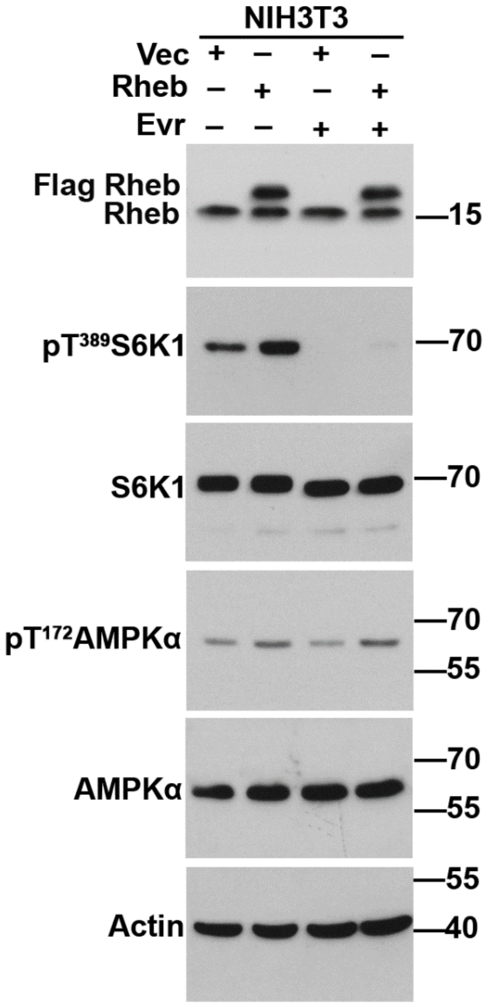

Figure S7. Role of mTORC1 in Rheb regulation of AMPK. Western blot analysis of Rheb, S6K1, $pT^{389}S6K1$, AMPKα, $pT^{172}AMPK\alpha$, and actin loading control in NIH3T3 cells transfected with Flag-Rheb cDNA for overexpression (Rheb), or vector control (Vec) and treated with or without 10 nM mTORC1 inhibitor everolimus for 24 hours.

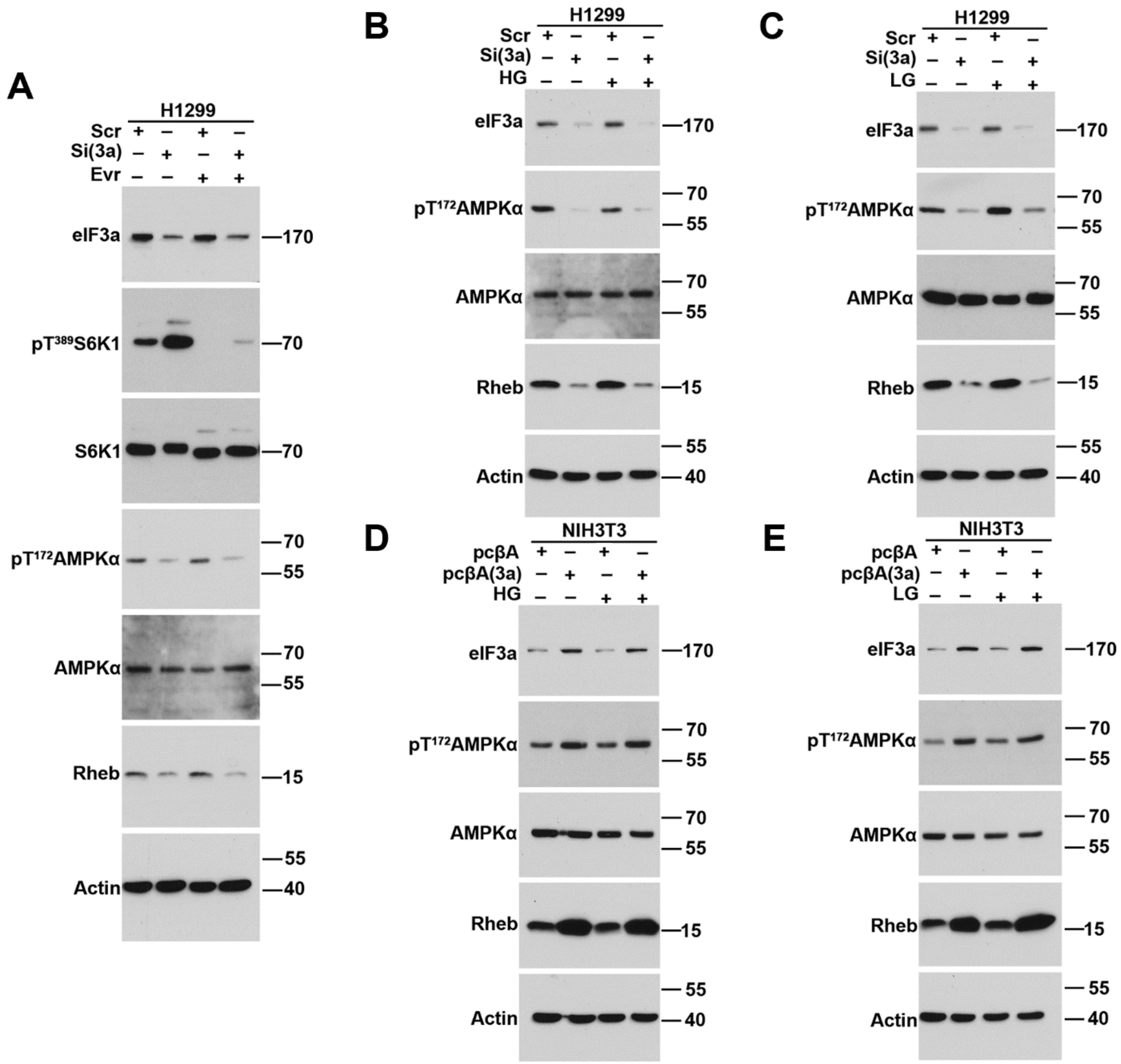

Figure S8. Role of mTOR signaling and glucose level in eIF3a regulation of Rheb and AMPK. (A). Western blot analysis of eIF3a, Rheb, AMPKα, pT^{172}AMPKα, S6K1, pT^{389}S6K1 and actin loading control in H1299 cells transfected with scrambled control (Scr) or eIF3a (Si(3a)) siRNA and treated with or without 10 nM mTORC1 inhibitor everolimus for 24 hours. (B-C). Western blot analyses of eIF3a, Rheb, AMPKα, pT^{172}AMPKα, and actin loading control in H1299 cells transfected with scrambled control (Scr) or eIF3a (Si(3a)) siRNA and cultured in the presence 25 mM (HG) (B) or 0 mM glucose (LG) (C) for 24 hours. (D-E). Western blot analyses of eIF3a, Rheb, AMPKα, pT^{172}AMPKα, and actin loading control in NIH3T3 cells with stable eIF3a overexpression (eIF3a) or harboring vector control (Vec) cultured in the presence of 50 mM (HG) (D) or 10 mM glucose (LG) (E) for 24 hours. The control normal glucose concentrations are 11 and 25 mM in RPMI1640 media for H1299 and DMEM for NIH3T3 cells, respectively.

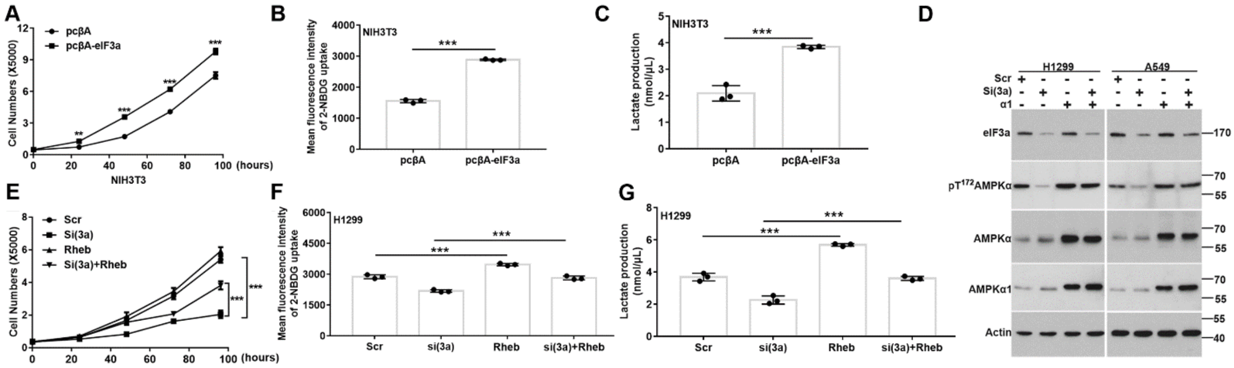

Figure S9. eIF3a and Rheb regulation of cell proliferation and glucose metabolism. (A-C). Proliferation (A), 2-NBDG uptake (B), and lactate production (C) in NIH3T3 cells with stable eIF3a overexpression (eIF3a) compared with control vector-transfected cells (Vec). (n=3, ***p*< 0.01, ****p*<0.001). (D). Western blot analyses of eIF3a, pT^{172}AMPKα, AMPKα, AMPKα1, and actin loading control in H1299 and A549 cells transfected with scrambled control (Scr) or eIF3a (Si) siRNA, or combination of both siRNA transfection and AMPKα1 cDNA for overexpression (α1). (E-G). Proliferation (E), 2-NBDG uptake (F), and lactate production (G) in H1299 cells transfected with scrambled control siRNA (Scr), eIF3a siRNA (Si(3a)), Flag-Rheb cDNA for overexpression (Rheb) or both eIF3a siRNA and Flag-Rheb cDNA. (n=3, ****p*<0.001).
